# Abdominal Cocoon Syndrome: A Rare Cause of Intestinal Obstruction

**DOI:** 10.7759/cureus.22929

**Published:** 2022-03-07

**Authors:** Joana Frazão, Ana Rita Martins, José Calado, António Godinho

**Affiliations:** 1 General Surgery, Hospital Prof. Doutor Fernando Fonseca, Lisboa, PRT

**Keywords:** adherences, encapsulation, sclerosing encapsulating peritonitis, cocoon, intestinal obstruction

## Abstract

Abdominal cocoon syndrome, also known as Sclerosing Encapsulating Peritonitis, is characterized by a fibro-collagenous membrane that involves abdominal viscera and it’s a rare cause of intestinal obstruction.

We present here two cases. Two male patients, 29 and 75 years old, were admitted to our emergency department with abdominal pain, vomiting, tender and painful abdomen but without changes in intestinal transit or peritoneal reaction. They were treated surgically and diagnosed with abdominal cocoon syndrome.

Patients with abdominal cocoon syndrome usually present with recurrent episodes of intestinal obstruction, which result from the compression of the bowel within the constricting cocoon. Most of the time, this clinical picture resolves with conservative measures, delaying the diagnosis. The definitive treatment consists of excision of the membrane with lysis of adhesions, which is usually reserved for more severe cases of obstruction.

This is a rare disease, where a high suspicion index is of paramount importance, especially considering that most of the diagnoses are made at the surgery.

## Introduction

Abdominal cocoon syndrome, also known as Sclerosing Encapsulating Peritonitis, is a rare entity and an extremely uncommon cause of intestinal obstruction. Being first detailed in 1978 by Foo et al. [1], it was mostly described in young adolescent girls, probably in relation to retrograde menstruation [2,3]. It consists of a total or partial encapsulation of the small bowel by a fibrous membrane with local inflammatory infiltrate leading to obstruction. Two variants exist: primary (idiopathic) and secondary, which is mostly seen in patients with a history of tuberculosis, neoplasm, beta-blockers use, peritoneal dialysis or previous abdominal surgery [4].

We hereby present two cases of idiopathic abdominal cocoon syndrome.

## Case presentation

Case 1

**Figure 1:**
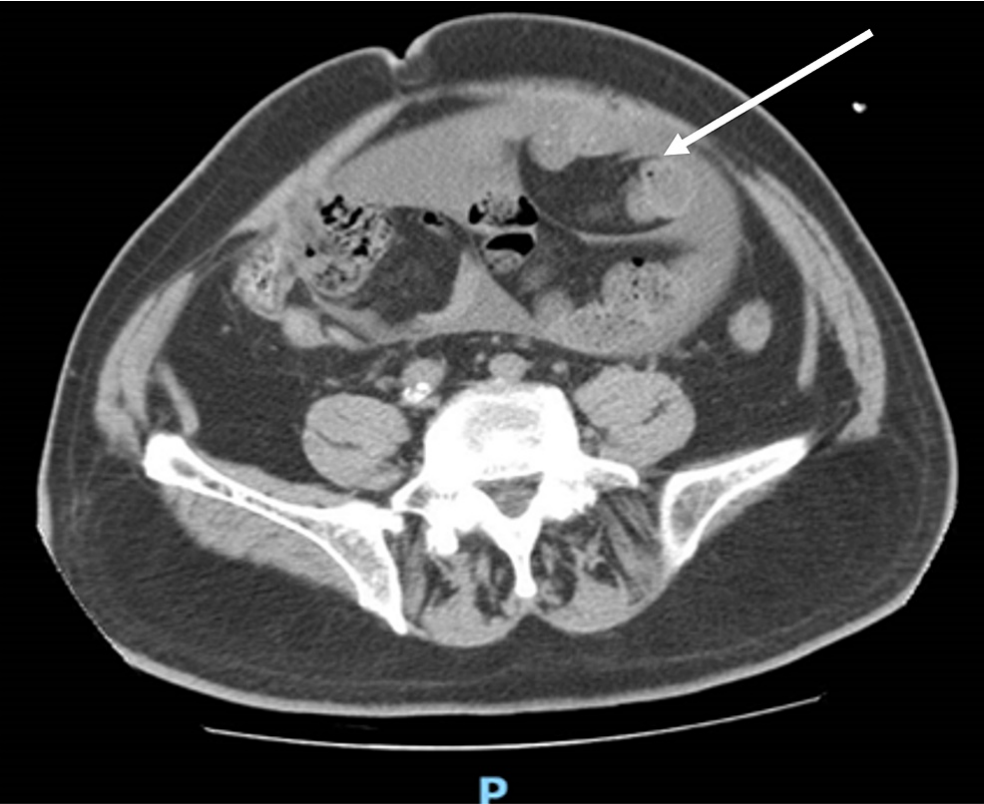
Coronal sections of abdominal CT images indicate small bowel dilation with fluid involving the bowel loops.

**Figure 2:**
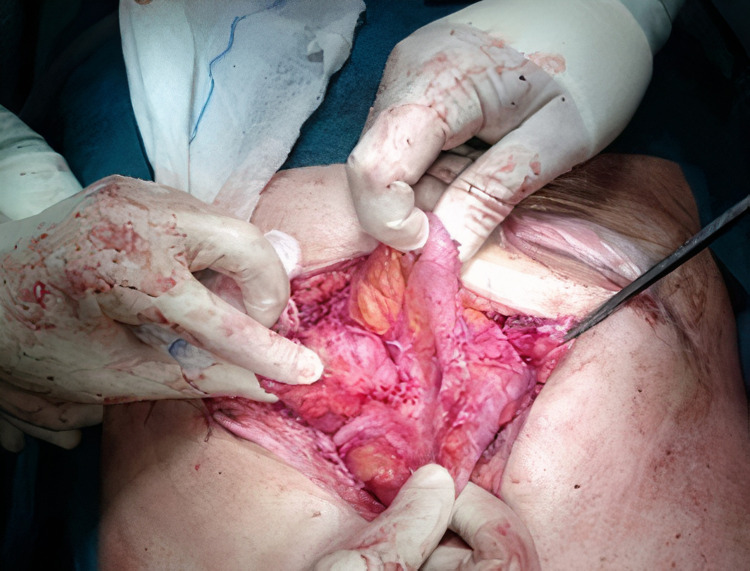
Intra-op image: small bowel involved by a “cocoon” like fibrous membrane.

A 75-year-old male patient, without past known diseases, presented with abdominal pain, nausea and vomiting, albeit with normal intestinal transit. He had a history of several past admissions to our Emergency Department with the same complaints that were solved with conservative measures. On clinical examination, the abdomen was tender and painful, but depressible and without peritoneal reaction. Abdominal CT scan (Figure 1) showed small bowel dilation, with fluid involving the bowel loops, suggesting an intestinal obstruction, that did not resolve with conservative measures. At laparotomy, the small bowel was involved by a “cocoon” like fibrous membrane (Figure 2). The section of this membrane resulted in a decompression of the entire small bowel. There was a segment of the ileum that was not viable, so an enterectomy and an ileocolostomy with both limbs brought together to the skin (Paul Mikuliz procedure) were performed. Histopathological analysis revealed intestinal necrosis as well as intense serositis of the fibrous membrane covering the bowel. On the tenth post-operative day, the patient was discharged. During the follow-up, the patient had several episodes of dehydration caused by the ileostomy. Thus, intestinal transit reconstruction with ileo-colic anastomosis was performed. After that, the patient evolved favorably.

Case 2

**Figure 3:**
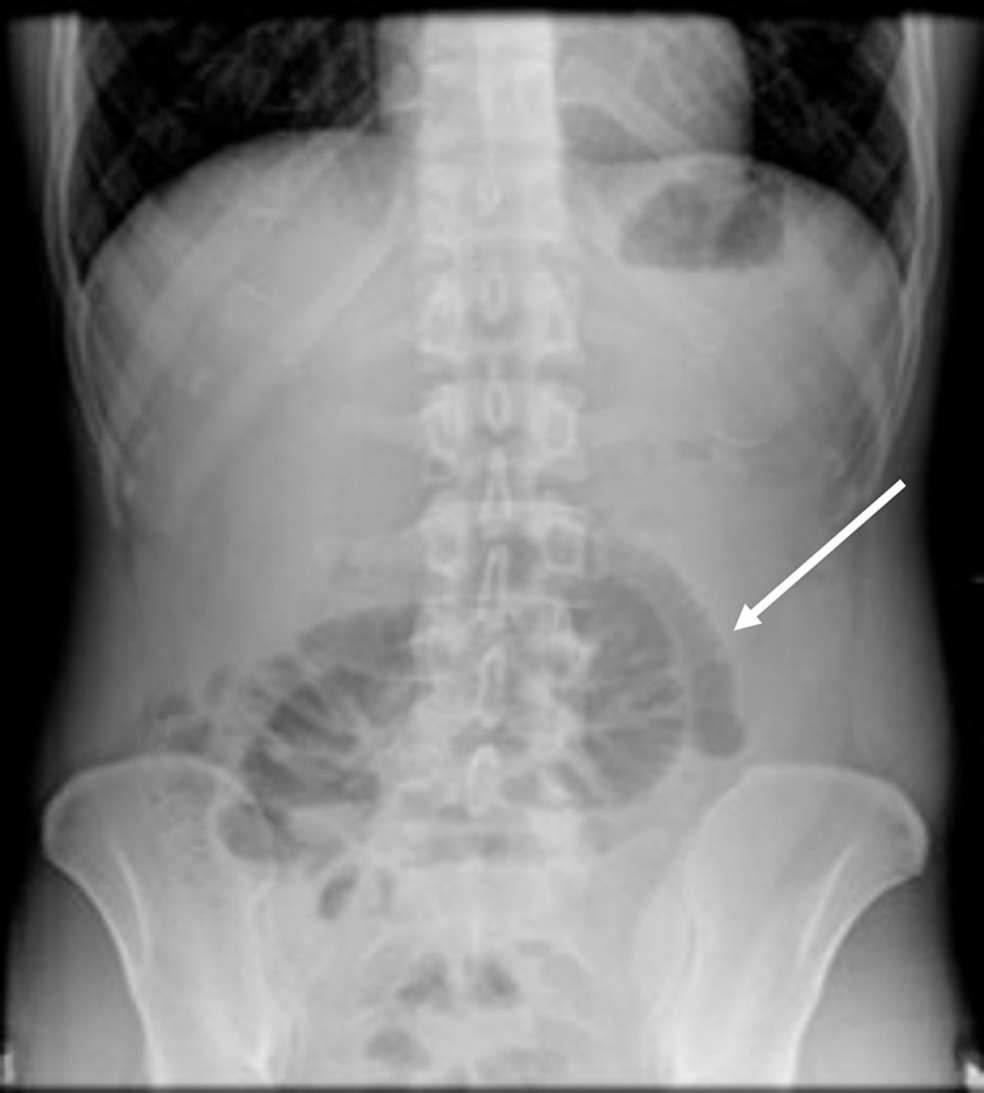
Air-fluid levels on X-ray showed a small intestinal obstruction.

**Figure 4:**
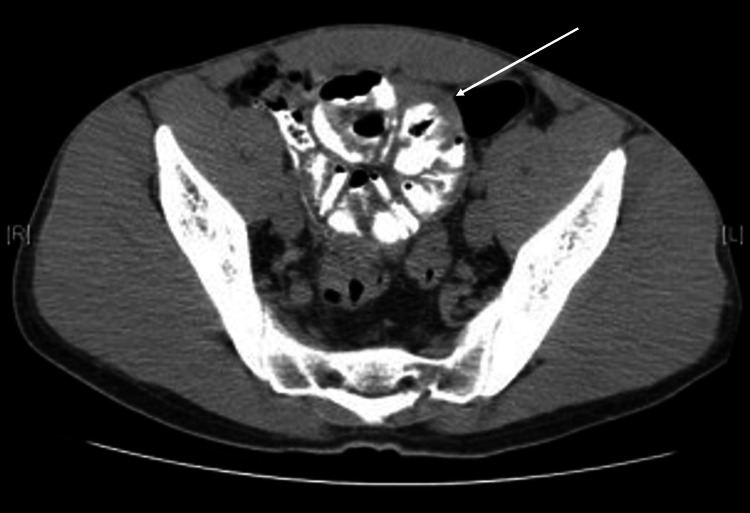
Coronal sections of abdominal CT images showed a part of small bowel embedded within a thin-walled fluid-filled sac-like structure.

**Figure 5:**
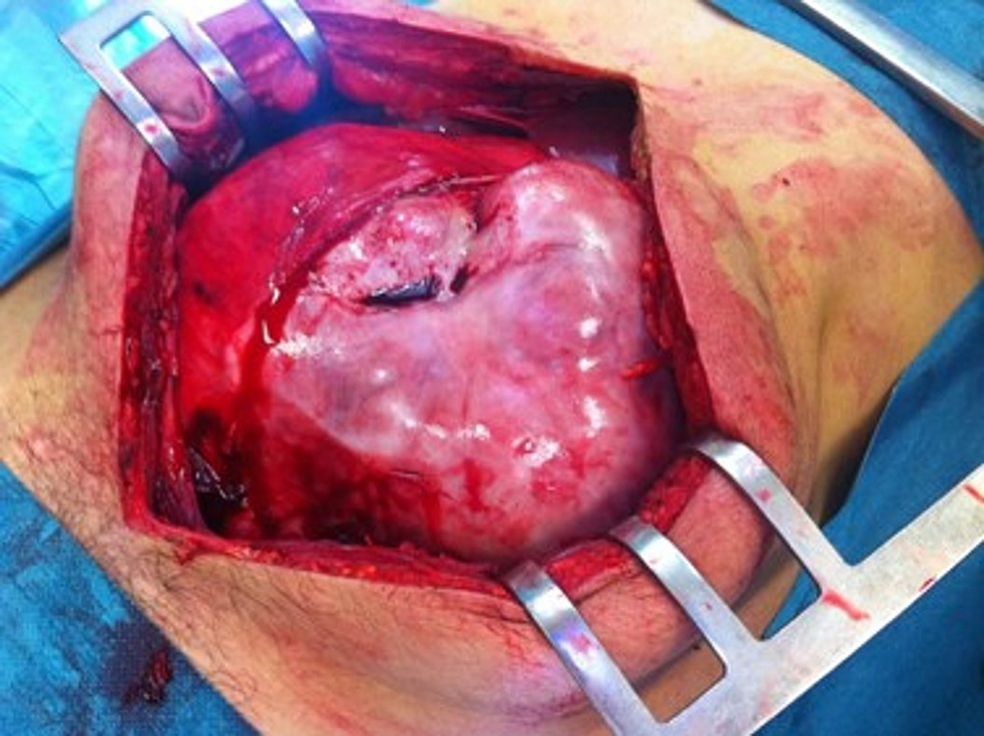
Intra-op Image: encapsulated part of the small intestine.

A previously healthy 29-year-old male patient presented with diffuse abdominal pain and vomiting, but with preserved bowel movement. He had a previous clinical history of several admissions to our Emergency Department with similar complaints that had initiated two years before. The abdomen was distended and painful but without peritoneal reaction. Plain abdominal X-ray (Figure 3) showed small bowel air-fluid levels and the abdominal CT scan (Figure 4) showed dilation of bowel loops with a minimal quantity of fluid present, suggesting an intestinal obstruction. This obstruction did not resolve with conservative measures, so the patient underwent exploratory laparotomy where an elastic membrane covering the entire small bowel was found (Figure 5). This membrane was cut and the small bowel loosened up, confirming its viability. The patient was discharged on the ninth post-operatory day, and after that he evolved favorably.

## Discussion

Abdominal cocoon syndrome, first described by Foo et al. in 1978, is characterized by a fibro-collagenous membrane which involves abdominal viscera, potentially leading to intestinal obstruction [1]. There are three types of abdominal cocoon: type I is partial encapsulation of the intestine; type II is complete encapsulation of the entire intestine; and type III is encasement of the entire intestine and other intra-abdominal organs (appendix, caecum, ascending colon and ovaries). Despite there being several known risk factors (tuberculosis, neoplasm, beta-blockers use, peritoneal dialysis and previous abdominal surgery), the etiology remains unknown in most cases [4]. Since our patients had none of those factors, we considered them both as an idiopathic case of abdominal cocoon syndrome.

Clinically, most patients present with recurrent episodes of abdominal pain and intestinal obstruction, secondary to kinking or compression of the bowel caused by the constricting cocoon [4,5]. This was demonstrated by both our cases who had several admissions with similar symptoms.

Although radiologic imaging plays an important role, it is not usually helpful in distinguishing abdominal cocoon syndrome from other intestinal obstruction-causing conditions [6]. Radiographs may show air-fluid levels and dilation of bowel loops. Abdominal CT scans may show intestinal obstruction, ascites and peritoneal or mesenteric thickening [7].

Preoperative diagnosis is difficult and, in most cases, the diagnosis is only established during exploratory laparotomy [5,8].

Management of abdominal cocoon is controversial. In patients that present with mild symptoms, conservative treatment, which includes nasogastric tube decompression, bowel rest and hydration, can be sufficient, but can delay the diagnosis. In patients presenting with severe signs of intestinal obstruction, the best approach consists of adhesiolysis and excision of the covering membrane, to free the entire small bowel [3]. Resection of the bowel may be necessary in cases where there is a vascular compromise, which happened in the first case described in this report.

The prognosis after surgery is usually excellent [3].

## Conclusions

Abdominal cocoon syndrome is a rare entity that can cause intestinal obstruction. Since most of the diagnoses are made intra-operatively, a high suspicion index is of paramount importance. In cases of mild obstruction symptoms, conservative treatment can be an option. Patients with severe intestinal obstruction need surgery to remove the entire fibrous membrane, which usually has an excellent prognosis and outcome.
